# Quantitative evaluation of fracture healing progression in rat femurs with modal damping factor and conventional methods

**DOI:** 10.3389/fbioe.2025.1697071

**Published:** 2026-02-06

**Authors:** Stavros Chalikias, George Anastassopoulos, John Sarris, Stefania Athanasopoulou, Malvina Orkoula, Christos Kontoyannis, Vassilis Kostopoulos, Spyridon Psarras, Nikolaos Papaioannou, Efstathios Chronopoulos, Ismene Dontas, Sofia Panteliou

**Affiliations:** 1 Orthopaedic Department, NHS Ayrshire and Arran, Trauma and Orthopaedics, University Hospital Crosshouse, Kilmarnock, United Kingdom; 2 Merchant Marine Academy, Aspropyrgos, Greece; 3 Department of Mechanical Engineering and Aeronautics, University of Patras, Patras, Greece; 4 Laboratory of Pharmaceutical Analysis, Department of Pharmacy, University of Patras, Patras, Greece; 5 Applied Mechanics and Vibrations Lab, Department of Mechanical Engineering and Aeronautics, University of Patras, Patras, Greece; 6 Laboratory for Research of Musculoskeletal System “Th. Garofalidis”, General Hospital of Attica “KAT”, School of Medicine, National and Kapodistrian University of Athens, Athens, Greece; 7 Laboratory of Machine Elements, University of Patras, Mechanical Engineering and Aeronautics, Patras, Greece

**Keywords:** modal, damping, fracture, healing, bone, animal model

## Abstract

Quantitative determination of bone fracture healing through objective measurements on the fracture area during healing phases is of paramount importance. In this study, simultaneous modal damping factor (MDF) testing was compared to peripheral quantitative computed tomography (pQCT), Raman spectroscopy (RS), and absorbed and fracture energy. MDF is a non-invasive index based on the model’s dynamic characteristics that applies vibration excitation. The method has been successfully applied as a structural integrity monitoring tool for defective conventional and advanced materials, including bones. We investigated whether MDF could identify when a functional and biomechanically adequately strengthened callus developed in osteotomized rat femurs. The measured property value intervals indicate that MDF correlates with all properties and detects the bone quality changes due to fracture and fracture healing with higher sensitivity than other methods. MDF monitors bone fracture healing and correlates with all parameters examined in a more accurate and sensitive way than conventional methods. Research findings support MDF as the most convenient of the methods examined for monitoring the bone fracture healing process.

## 1 Introduction

Quantitative determination of bone fracture healing through objective measurements representing the three bone healing phases (soft callus, solid callus, and remodeling phase) is expected to lead to valuable information (Marsell and Einhorn, 2011).

Conventional methods for evaluation of bone fracture healing are X-rays, computed tomography (CT), magnetic resonance imaging (MRI), and peripheral quantitative computed tomography (pQCT). Raman spectroscopy (RS), micro-CT, and three-point bending (3PB) are additional tools used for experimental purposes (Clark and Badea, 2014).

MDF is a non-invasive, user-friendly, non-expensive method that has been proven to directly relate to strength, accounts for structural changes, and is based on an assessment of a model’s dynamic characteristics by applying vibration excitation (Dimarogonas, 2001). This method was successfully applied as a structural integrity monitoring tool on defective structures made of conventional and advanced materials in order to monitor crack propagation and changes in porosity (Panteliou and Dimarogonas, 1997; Panteliou et al., 1999; Panteliou and Dimarogonas, 2000; Panteliou et al., 2001; Panteliou et al., 2004; Panteliou et al., 2009a; Panteliou et al., 2009b; Anastassopoulos et al., 2010; Panteliou et al., 2012; Pantel et al., 2012). The method has also been applied to bones (Chalikias et al., 2021) to monitor changes in porosity, thus enabling assessment of metabolic bone diseases, especially osteoporosis. The role of sensors has been well-established in the literature. Sensor devices have been constructed and used in various disciplines (synthesis and catalysis) for monitoring parameters, like response time, analytical ranges, and operating temperatures during reactions (Shah et al., 2022; Shah et al., 2025).

In this original and prototype study, we extended our previous research to investigate the hypothesis that MDF may be used as an auxiliary, reliable, and competitive tool for quantitatively monitoring the process of bone fracture healing in rat femurs compared to conventional methods.

## 2 Materials and methods

### 2.1 Animals

We used 48 6-month-old male Wistar rats (Institut Pasteur Hellenique, Athens, Greece) raised conventionally with no previous experimental procedure and a 1 month acclimatization period, randomly allocated to four groups (A, B, C, and D), each with 12 rats, following a sample size estimation using G power, with each rat representing an experimental unit. Experimental procedures were conducted according to the EU Directive 2010/63/EU and licensed according to Greek PD 56/13 “for protection of animals used for experimental purposes” (protocol license no. 3003/15.05.2014).

A transverse osteotomy was performed at the rat femoral midshaft with a Gigli wire saw. The fracture remained fixed by an intramedullary RatNail throughout the experimental period. After osteotomy, 12 rats each time were euthanized at postoperative weeks 6, 8, 10, and 12, corresponding to Groups A, B, C, and D of the healing process (Chalikias et al., 2021). In adult rats, fracture union (callus) is achieved within 10 weeks (Ekeland et al., 1982; Mølster, 1984). Then, both femurs were harvested by joint disarticulation, without affecting the fracture callus, and the intramedullary nail was withdrawn. Potential fracture non-union observed at euthanasia was considered *a priori* an exclusion criterion. One observed rat was replaced; each group completed the study time with 12 rats.

In the following, indices R-L refer to right (operated) and left (control) femurs. Indices Pre-Post refer to pre- and postoperative conditions.

### 2.2 Measurements - All femurs (from 12 animals of each of the four groups) underwent the following measurements

#### 2.2.1 Bone density

The bone density of both femurs was determined by pQCT bone density measurements (Norland Stratec XCT 2000). These measurements were acquired 1 week before osteotomy surgery (Pre) and *ex vivo* (Post).

##### 2.2.2.1 RS

A micro-Raman spectrometer (i-Raman Plus, B&W Tek, USA) with a 785 nm laser line was used. Spectra were collected from different spots of the bone periosteum (fracture vicinity) and endosteum (fracture section), with a beam focused through a ×50 objective lens. Three spectral regions of interest were isolated: a) prolines (800–900 cm^−1^), b) apatite (900–990 cm^−1^), and c) amide I envelope (1,590–1,730 cm^−1^). Analysis included baselining band deconvolution and curve fitting (PeakFit^©^ v4.0, Jandel Scientific, San Rafael, CA). The intensity of the primary phosphate band (PO_4_^3−^,*v*_1_) at 959 cm^−1^ and the matrix bands at 855 cm^−1^ (proline), 875 cm^−1^ (hydroxyproline), and 1,668 cm^−1^–1,685 cm^−1^ under the amide I envelope were measured. The following metrics were calculated: mineral-to-matrix ratio (MMR) [I (959 cm^−1^)/{I (855 cm^−1^)+I (875 cm^−1^)}] and cross-linking ratio (CLR) [I (1,668 cm^−1^)/I (1,688 cm^−1^)], which corresponds to the non-reducible to reducible cross-collagen link ratio.

##### 2.2.2.2 X-ray microtomography

Samples were scanned using a micro-CT scanner (SkyScan 1174, Bruker, Germany). Settings: 50 KV tube voltage and 800 μA current, Al filter (1 mm thickness), 180° scan with 0.5° rotation step. In each image, the average of five frames is shown. Samples were placed in an airtight plastic tube for scanning to prevent exposure to the atmosphere. Every scan lasted 5 h. Image reconstruction was done using appropriate software (NRecon, Bruker, Germany).

#### 2.2.2 Βiomechanical tests

Biomechanical tests serve as the gold standard for strength measurements. Charpy impact and 3PB tests were applied to measure the flexural strength and fracture energy of the bone samples *ex vivo.* From the 3PB test, total fracture energy (elastic + inelastic) was calculated. This value is referred to as absorbed energy, to distinguish it from dynamic (Charpy) fracture energy.

Tests were performed according to ASTM standards, whenever possible, or using dimensions close to the relevant standard (ASTM D790-99, D6110-97).

Specimens were cut to the requested dimensions using a diamond saw under a wet environment and preserved under appropriate storage conditions.

#### 2.2.3 MDF

A dedicated device was built to conduct MDF measurements on R-L femurs 1 week before the osteotomy and after euthanasia (Pre-Post). Fracture callus quality was assessed from the correlation between the data from MDF and established methods (pQCT, RS, and biomechanical tests).

## 3 Results

### 3.1 Bone density

Total bone mineral density data [kg/m^2^], Pre-Post L-R, were collected with pQCT. Post measurements were taken on five femur sections, 1–5. Section 3 coincides with the osteotomy section for callus formation monitoring.

### 3.2 RS

Collagen network measurements were acquired *ex vivo* for R-L femurs (all specimens and times).

#### 3.2.1 Raman metrics

The mineral-to-matrix ratio (MMR) is correlated to bone mineralization. It starts from a zero value for an unmineralized collagen network and increases to a maximum for mature healthy bone. Decreased values are recorded in pathological cases (osteoporosis) or in aged bone with reduced mass (Mandair and Morris, 2015).

The collagen linking ratio (CLR) is associated with collagen network quality. It is defined as the ratio of non-reducible to reducible cross-links between collagen fibers. A newly formed collagen network exhibits an immediate premature maximum, which reduces toward maturity value (Mandair and Morris, 2015; Tarnowski et al., 2002).

#### 3.2.2 Raman metrics for fractured femurs through time; correlation to MDF values

**FIGURE 1:**
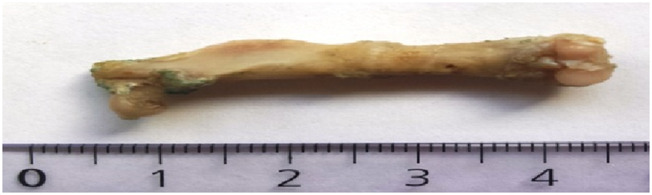
Soft callus on osteotomized femur (subgroup I) [cm].

**FIGURE 2:**
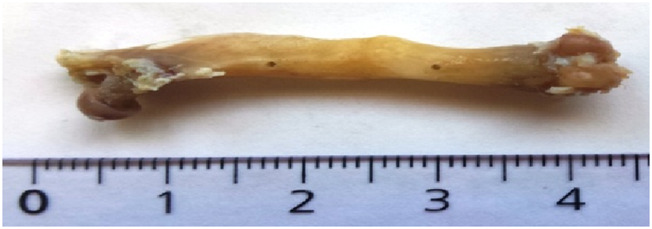
Hard callus on osteotomized femur (subgroup II) [cm].

**FIGURE 3:**
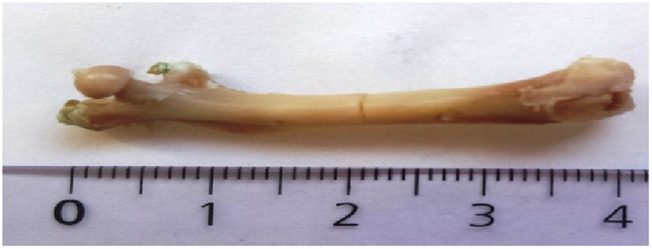
No callus on osteotomized femur (subgroup III). Crack obvious [cm].

The progressive change of the two Raman bio-indices, MMR and CLR, with time, for the four animal groups (A, B, C, and D, which are 6, 8, 10, and 12 weeks after osteotomy, each with 12 rats) was monitored and correlated to MDF changes. Groups were subdivided into three subgroups (I, II, and III) according to callus type (soft, hard, or no callus) (Figures 1–3).

### 3.3 Βiomechanical tests

Charpy impact and 3PB tests were applied to determine femur fracture properties (Groups A, B, C, and D, each with 12 rats) under mechanical loading. Two test speeds were incorporated, utilizing the appropriate equipment. A Charpy impact test machine was used for low-velocity fracture tests, and a universal servo-hydraulic testing machine was used for pseudo-static 3PB tests. The absorbed impact energy was measured as the first test type, and force-displacement was measured for the latter ones. Half of each group underwent the absorbed energy test, while the other half underwent the fracture energy test.

Absorbed energy is the amount of energy an object receives during a force impact. It represents the work done by the impact force to deform the object, is defined as the portion of the kinetic energy or the work done by the impact force that is converted into deformation, and refers to the energy the object can absorb before fracture.

Fracture energy is the energy needed to break an object completely.

#### 3.3.1 Results of experimental procedure

##### 3.3.1.1 Charpy impact

**FIGURE 4:**
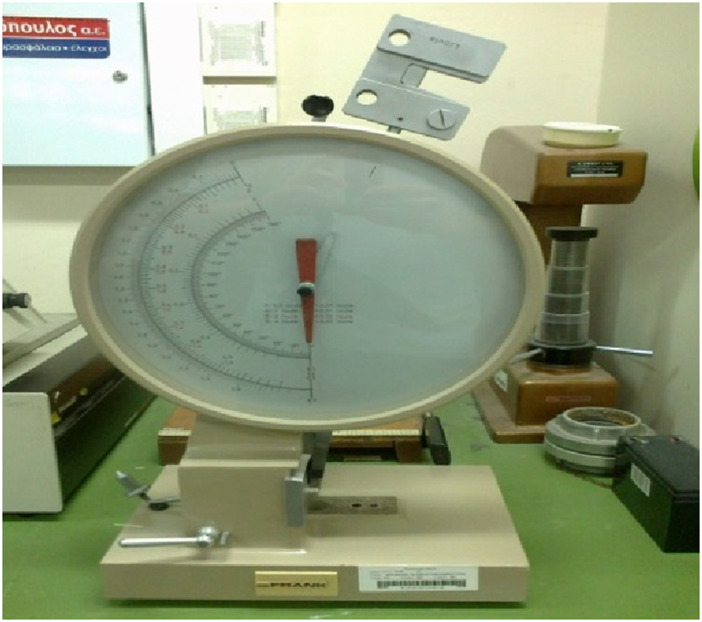
Charpy test machine.

**FIGURE 5:**
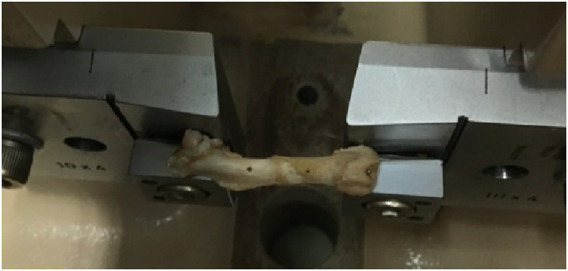
Specimen placement.

A typical Charpy/Izod test machine was utilized (Figure 4). A loading head rated to 1,000 mJ maximum energy was placed on the pendulum. Femurs were placed on the support, ensuring maximum possible stability (Figure 5). Bone orientation was kept the same for all specimens.

After specimen placement, the loading head was raised to the default height and released. The energy absorbed by the femur fracture was recorded manually.

##### 3.3.1.2 Three-point-bending

**FIGURE 6:**
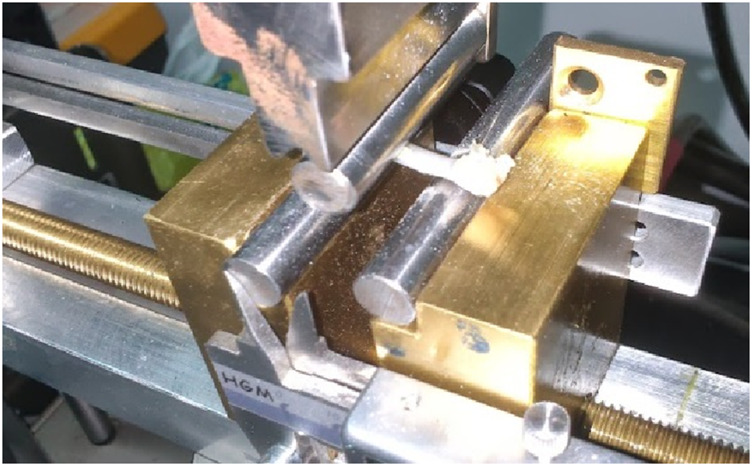
Test jig.

**FIGURE 7:**
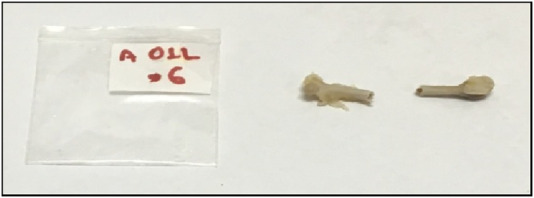
Fractured specimen A01L6.

Femurs were subjected to pseudo-static loading to determine flexural strength. The test jig (Figure 6) comprises two supports and a loading nose (radius 5 mm). Span length was set as long as possible (14 mm). Fractured specimen A01L6 is shown in Figure 7.

**FIGURE 8:**
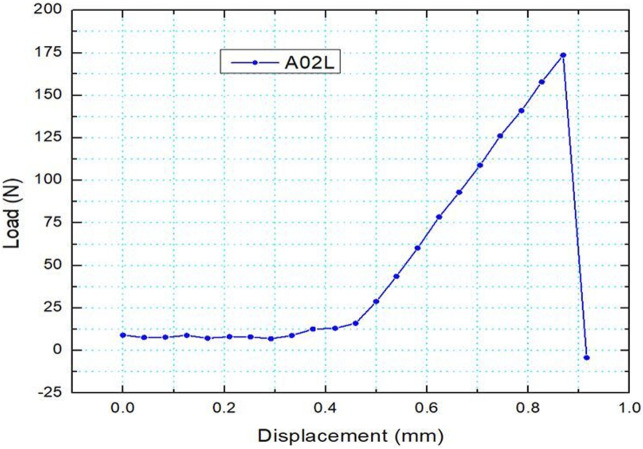
Load vs. Extension (Specimen A02L).

Displacement rate was set at 50 mm/min. The test was carried out until specimen failure, thus extracting load–displacement curves. Except for the maximum (failure) load, fracture energy can be calculated using the work of force acting on the femur (area beneath the load–displacement curve). A typical load–extension graph is shown in Figure 8 (Specimen A02L). In some cases, the femur was broken, or the load-to-failure was so low that the energy value could not be calculated.

### 3.4 Modal damping factor

**FIGURE 9:**
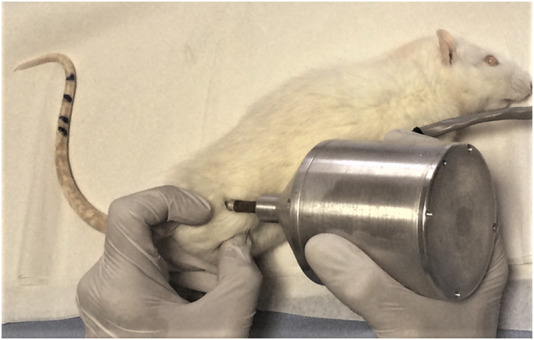
Device for MDF measurement.

These tests were carried out on R-L femurs 1 week before osteotomy and after euthanasia. Pre(L), Pre(R), Post(L), and Post(R) MDF values were obtained for each of the specimens (A1–A12, B1–B12, C1–C12, and D1–D12). A dedicated device was built for this purpose (Figure 9). The sensing rod of the device was placed in such a way as to ensure firm contact with the point to be measured and at as constant an angle as possible. The device was used at room temperature to avoid the effect of environmental changes. Before application, the device was tested on specimens of known MDF values for calibration purposes. Each MDF value is the average of 10 consecutive measurements.

## 4 Statistical processing

**FIGURE 10:**
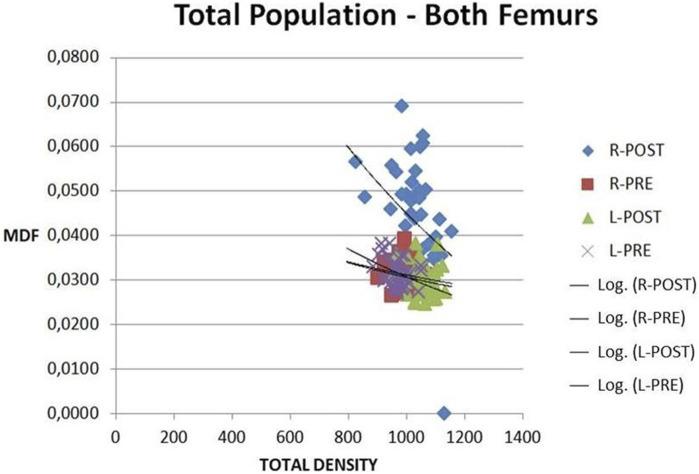
MDF vs. TOTAL DENSITY. All subjects, all time points. L-R femurs, Pre and Post.

The MDF and total density correlations for R-L femurs, Pre-Post, at all time points are shown in Figure 10. Postoperative sets of values correlate as expected. Post MDF is clearly higher than Pre. This represents damaged bone quality due to the osteotomy. Differences in the MDF range are shown more clearly than in the total density one. MDF sets of values for the L femur Pre-Post and the R femur Pre lie in the same range area (0.022–0.038), which is explainable, given that there are practically no biological or other substantial changes. R femur Post values are clearly distinct from the R femur Pre and L femur Post sets (0.03–0.07) and are close to the damaged structure values, showing a clear distinction from the initial sets. MDF detects changes due to surgery more sensitively than total density. Total density values are in the same range (900–1,150) with no distinction. A few values out of the systematic range may be considered as false measurements. MDF is a dimensionless number taking values in the interval (0, 1) with higher values corresponding to damaged structure quality and vice versa.

MDF is a physical net value index expressing the quality of the whole structure, in the specific case, the fractured bone. During the bone healing process, the only physical characteristic that changes is the bone density as soft cartilaginous tissue becomes mineralized bone. Eventually, MDF accounts for this specific change, thus expressing the bone quality levels.

**FIGURE 11:**
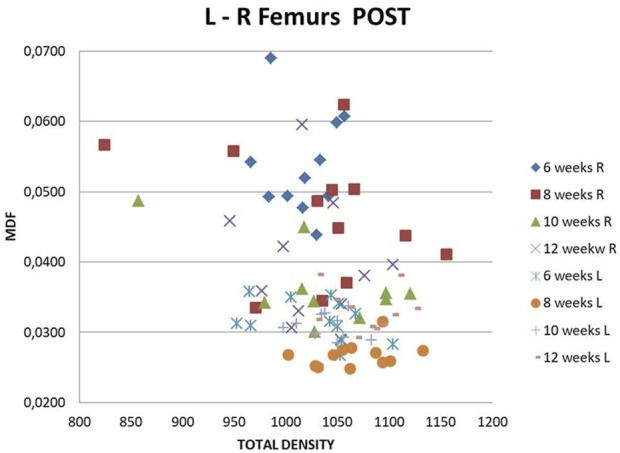
MDF vs. TOTAL DENSITY. All subjects, all time points, L-R femurs Post.

R-L femur bone quality improvement (callus formation) during consecutive time points (6, 8, 10, 12 weeks) is presented in Figure 11. Postoperative MDF and total density values correlate as expected, with MDF decreasing and total density increasing with time. Differences appear in the MDF range more clearly than in the total density one. R femur Post values lie far from the other sets. MDF detects the changes due to surgery more intensively, something that does not happen with total density. It is seen that all total density values are in the same range. Callus formation follows an increasing trend for weeks 6, 8, and 10. For week 12, a slightly inverse trend is observed (Tarnowski et al., 2002; Morgan et al., 2014).

**FIGURE 12:**
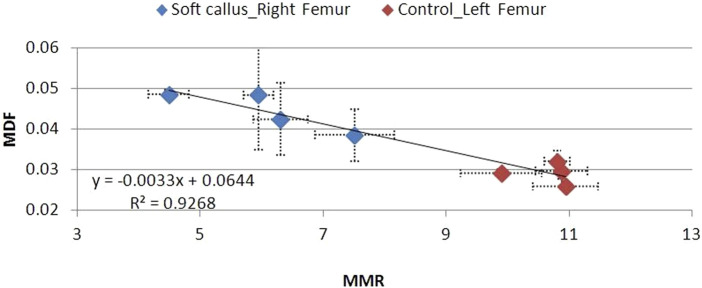
MDF to MMR values for animal Groups A–D exhibiting a soft callus, compared to non-osteotomized controls.

**FIGURE 13:**
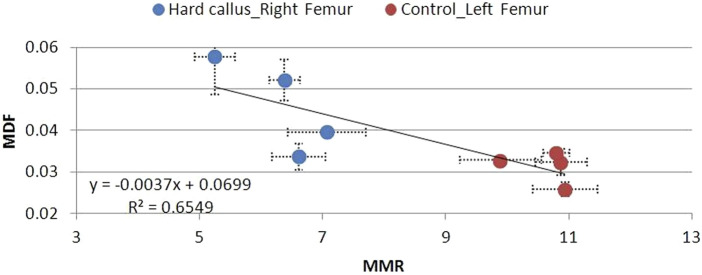
MDF to MMR values for animal Groups A–D exhibiting a hard callus, compared to non-osteotomized controls.

**FIGURE 14:**
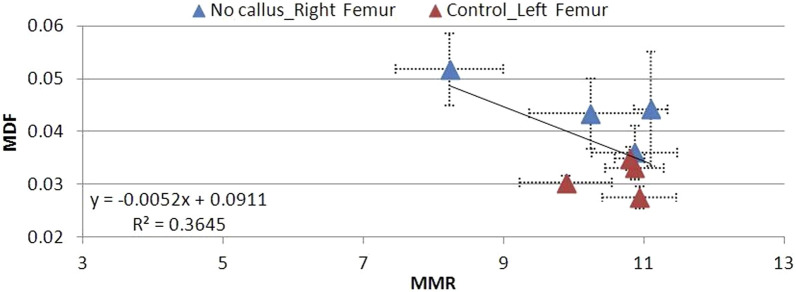
MDF to MMR values for animal Groups A–D exhibiting no callus, compared to non-osteotomized controls.

The L control femurs were characterized by low MDF and high MMR values, which do not vary significantly between 6 and 12 postoperative weeks (Figures 12–14). High MDF values accompanied by low MMR values were recorded for R femurs, 6 weeks after fracture (Group A), regardless of the degree of healing (soft, hard, or no callus). Next, MDF decreased and MMR increased at the same time, moving toward control values. Differences were noted in the fracture healing process. For animals that exhibited a soft callus, 6 weeks after fracture (Group A), a discrepancy of 64% in MDF and 59% in MMR compared to the control was recorded (Figure 12). The two parameters changed almost linearly through time, but 12 weeks after fracture, were no less than 30% in MDF and 31% in MMR, different from control values. Animals with a hard callus showed better mechanical behavior with time (Figure 13). MDF values were the worst in the first 6 weeks (Group A) after surgery, 95% higher than control, but rapidly progressed. At the 12th post week, Group D almost reached control values (15% difference). Unfortunately, the mineralization of the hard callus was not that successful, as the MMR of Group D was 39% away from the control. For animals following the progressive crack disappearance model (no callus), the best healing results were recorded 6 weeks after fracture. In this case, the MDF differed by 56% and the MMR differed by 24% from the control. At the 10th week, both parameters reached control values (Figure 14).

### 4.1 Result verification with X-ray microtomography

**FIGURE 15:**
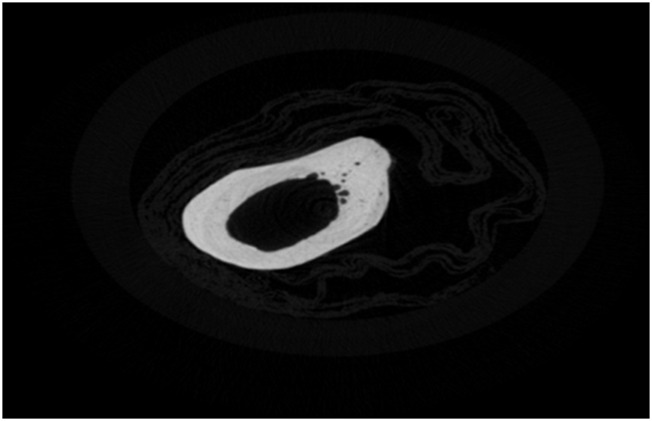
X-ray microtomography section of the L femur, 12 weeks after fracture of the R femur (Group D).

**FIGURE 16:**
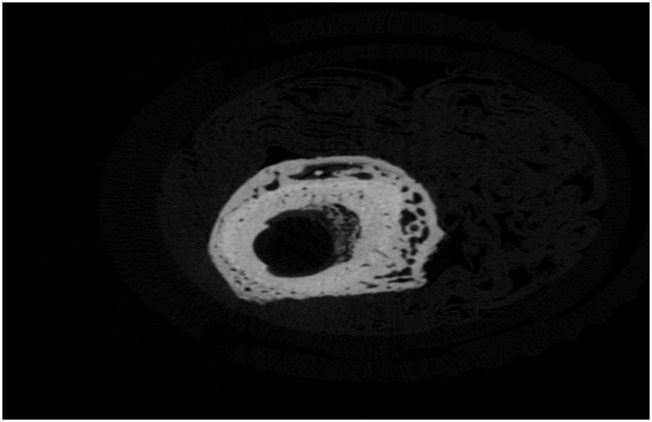
X-ray microtomography section near the crack of the R femur (Group D) exhibiting a soft callus.

**FIGURE 17:**
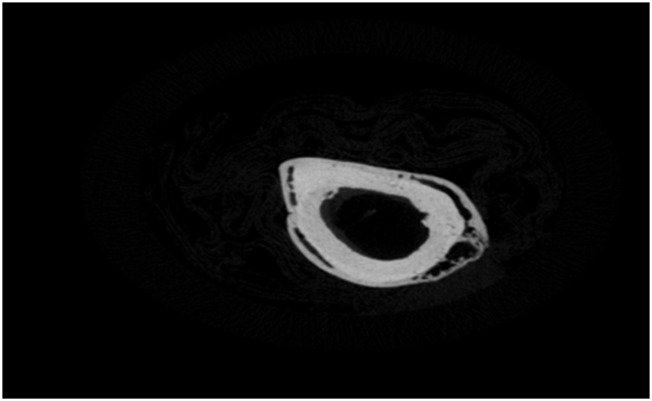
X-ray microtomography section near the crack of the R femur (Group A) exhibiting a hard callus.

**FIGURE 18:**
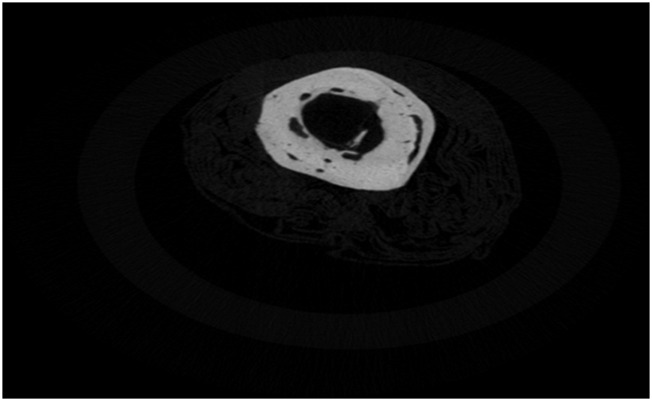
X-ray microtomography section near the crack of the R femur (Group D) exhibiting no callus.

The previously described healing characteristics were cross-validated with X-ray microtomography. The L femurs, 12 weeks after fracture induction on R femurs, exhibited a uniform and highly mineralized structure with limited porosity (Figure 15). This agrees with the previously shown min MDF and max MMR values. The fibrous network covering the femur around the crack, 12 weeks after fracture (Group D), is obvious in the section shown in Figure 16, for the R femur with a soft callus. Callus mineralization is less than that of the cortical bone, which explains the reduced MMR value compared to the control. Overall, the porous and inhomogeneous structure goes well with increased MDF. The hard callus that some femurs exhibited even in the sixth postoperative week stands well off the control (Figure 17). The lack of cohesion of the reformed bone around the crack with the pre-existing cortical bone is obvious and responsible for the MDF discrepancy between the healing bone and the control. X-ray microtomography of the femur, 12 weeks after fracture, where callus has been absorbed, reveals the bone remodeling process (Figure 18). Incoherent hard callus is almost absorbed. Mineralization has progressed satisfactorily, leading to increased MMR. The bone structure is still porous with lacunae, which justifies the MDF discrepancy with the control.

### 4.2 Collagen cross-linking ratio to MDF

**FIGURE 19:**
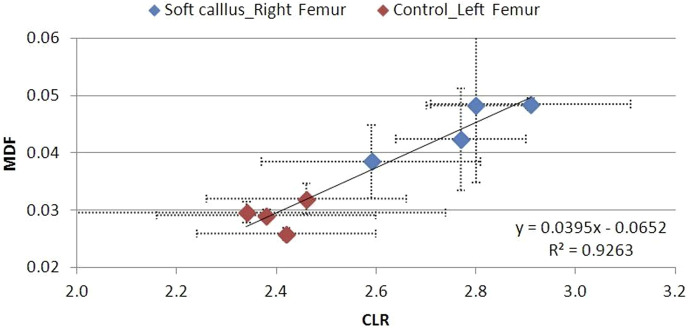
MDF to CLR values for animal Groups A–D exhibiting a soft callus, compared to non-osteotomized controls.

**FIGURE 20:**
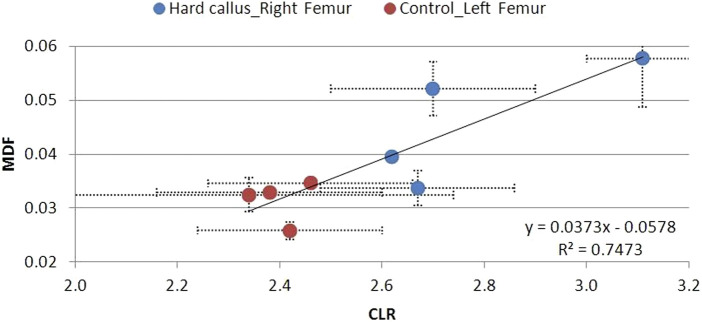
MDF to CLR values for animal Groups A–D exhibiting a hard callus, compared to non-osteotomized controls.

**FIGURE 21:**
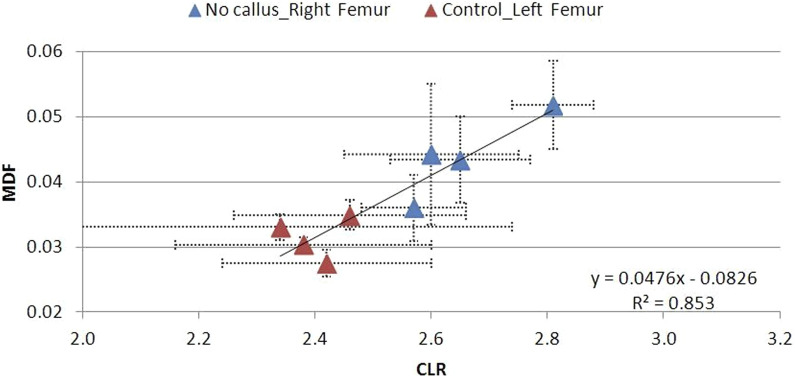
MDF to CLR values for animal Groups A–D exhibiting no callus, compared to non-osteotomized controls.

The MDF–CLR correlations over time for the soft, hard, and no callus subgroups are shown in Figures 19–21. CLR had a steep increase shortly after fracture (not shown) and decreased afterward, with no remarkable differences between the callus subgroups.

**FIGURE 22:**
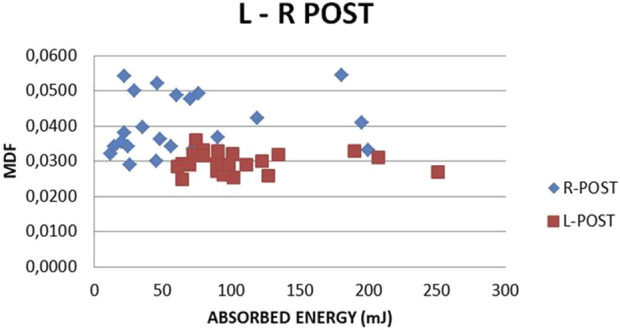
MDF vs. ABSORBED ENERGY. All subjects, L-R Post.

**FIGURE 23:**
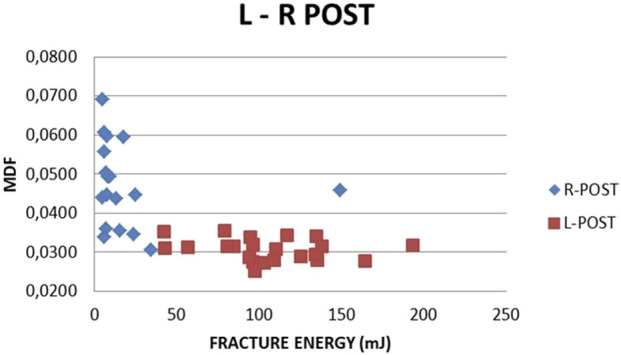
MDF vs. FRACTURE ENERGY. All subjects, L-R Post.

L-R Post MDF vs. absorbed and fracture energy correlations are seen in Figures 22, 23. A clear distinction between the two sets of values (L-R) is noticed. Smaller absorbed or fracture energy is needed to break R bones than L bones. This is explainable, given that the R bone quality was damaged due to the previous fracture. For L femurs, the absorbed energy value range (60, 250) and an MDF range (0.024, 0.037) are noticed (Figure 22). The relevant R values lie in the intervals (15, 200) and (0.029, 0.055). In the L femur, the fracture energy value range is (48, 200), and the MDF range is (0.022, 0.038), while for the R femur, the relevant ranges are (10, 40) and (0.03, 0.069) (Figure 23). The absorbed and fracture energy L values do not express reality because these values are expected to be almost constant due to steady conditions, whereas the MDF L values present a much lower scatter. Hence, MDF represents the evolution after surgery more realistically.

**FIGURE 24:**
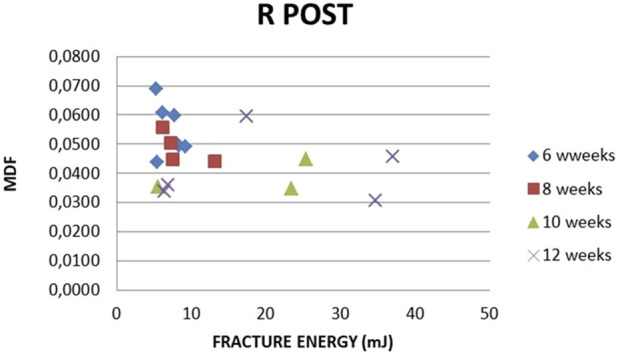
MDF vs. FRACTURE ENERGY. All subjects, all time points, R, Post.

**FIGURE 25:**
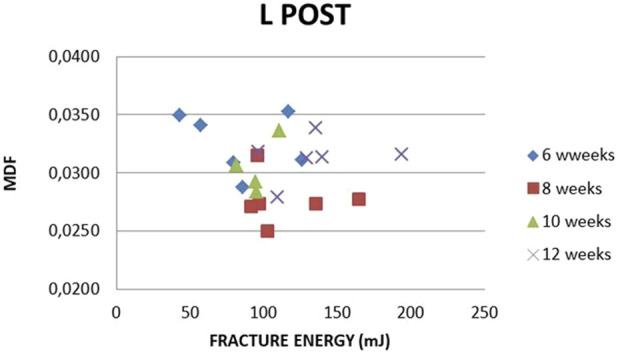
MDF vs. FRACTURE ENERGY. All subjects, all time points, L, Post.

The R-L Post MDF to fracture energy correlation with time is presented in Figures 24, 25. The fracture energy needed to break R femurs is increasing with time, while MDF is decreasing, both corresponding to callus formation. Unexplainable L Post fracture energy variation is noticed, while MDF remains practically stable. This is valid for weeks 6, 8, and 10. After week 10, a slight inverse trend is noticed.

## 5 Discussion

In this study, pQCT, RS, absolute, and fracture energy data were compared with MDF values, all acquired on the fracture area of osteotomized rat bones, to monitor bone fracture healing.

pQCT was applied to measure the bone mineral density (BMD) of the osteotomy area. pQCT is inappropriate for bone strength estimation and measurement, as its ability to assess bone strength is based on calculation. In our study, BMD did not show statistically significant differences between the rat groups examined at different time points for R osteotomized femurs, whereas MDF clearly detects the fracture healing as derived from Figures 10, 11.

### 5.1 MDF–MMR

There is a strong correlation between MDF and the RS bio-index, MMR. Healthy mature bone exhibits low MDF and high MMR values, as expected. During fracture healing, MDF values follow the type of callus formed, that is, the curing stage. Bone healing is accomplished by the formation of soft cartilaginous callus at the fracture site, made up of new connective tissue, to bridge broken bone fragments. At this stage, the MDF and mineralization Raman index indicate a worse structure. X-ray microtomography verifies the presence of spongy bone of low MMR and increased porosity (high MDF). In the second stage, this primary callus is transformed into a hard bony formation, the hard callus. Then, MDF decreases sharply as expected for higher structural integrity. Finally, the newly formed (woven) bone is remodeled, the callus is resorbed, and compact bone is added. This was ascertained in X-ray micro-images and accompanied by the closest-to-control MDF and MMR values. CLR, in all three stages, exhibits comparable values and changes.

### 5.2 MDF values reveal healing progress

The MDF values of fractured femurs clearly show structural quality improvement with time. There is a connection to the healing stage and success. At the 10th or 12th postoperative week (subgroups C and D), a decrease in MDF is noted only when the callus is hard or resorbed. If the callus remains soft and unmineralized at the previously mentioned time, the MDF does not approximate the values of healthy controls, thus expressing the structural loss. In the no callus cases (6 weeks after fracture), healing is not complete, and strong bone has not formed, as shown by low MMR, in mCT cross sections, and by MDF, which remains elevated, as expected. All the above can be seen in Figure 11 and Figures 12–14.

Pre-MDF mean L-R values coincide, Pre-Post L values also coincide, while Pre-Post R values are different, expressing evolution of the bone quality of the operated femurs compared to the condition before surgery, as expected (Figure 10). The improvement of bone quality with time is clearly expressed in the MDF Post L-R with time graphs (Figure 11). The R-L, Pre-Post total density values lie almost in the same range and do not enable an accurate diagnosis. Post MDF and total density values correlate for R Femur with the relevant Pre ones. Namely, R-Post MDF is clearly higher than R Pre and represents the damaged bone quality on account of the osteotomy. As the healing process progresses, the callus formed is reinforced due to mineralization. Approaching 10–12 weeks, the callus advances into the remodeling phase, the stage where the so-called “woven bone” is gradually replaced by mature lamellar bone. This transition is achieved by resorption, at least in part, of the previous structure. A small increase in MDF values documented at this point of the study can be ascribed to the above phenomenon. (Note: Lower MDF values correspond to healed bone, while higher values correspond to fractured bone. MDF values lie in the interval (0, 1), with higher values representing structural damage and vice versa.)

Differences in the MDF range are more obvious than differences in the total density range. Specifically, L Pre-Post and R-Pre MDF values lie in the same range, which is explainable, given that there are practically no biological or other substantial changes for these cases. R-Post MDF values are different from the Pre values, close to the damaged structure values, thus expressing a clear distinction from the initial sets. All total density values are in the same range with no distinction. A few values out of the systematic range may be considered as false measurements. Hence, MDF detects the changes due to surgery in a more sensitive and realistic way than total density, thus enabling objective monitoring of bone fracture healing and detailed diagnosis.

The L-R Post correlations between MDF with absorbed and fracture energy are seen in Figures 22, 23. A clear distinction between the two sets of values is noted. In addition, a very low amount of absorbed or fracture energy is needed to break the fractured bone (R) compared to the control bone (L). This is correct given that the L femur is integral and the R femur is fractured; thus, R has lower strength than L.

The fracture energy needed to break the R femur increases with time, while MDF decreases. Both findings correspond to callus formation. Unexplainable variation in fracture energy for the L femur is noticed, while MDF at the same time remains practically stable. These comments apply to weeks 6, 8, and 10. From then on, a slight inverse trend is noticed due to local micro-changes. Hence, MDF expresses the bone structural integrity after surgery in a more realistic way compared to absorbed and fracture energy.

After biomechanical testing, SEM inspection of fractured surfaces was applied for detailed identification of fracture mechanisms. We intend to use the concluded properties in bone finite element models in order to better simulate load transfer mechanisms through the fracture healing site. We will then compare the analytical and experimental results for analysis validation and modeling process verification. All finite element models will be based on real bone geometry that will be determined based on relevant CT scans and bone geometry reconstruction using Materialise Mimics software.

This study aimed to examine whether the MDF method can be used to monitor bone fracture healing and identify when a functional and biomechanically adequate strong callus forms in osteotomized rat bones. Simultaneous testing of MDF compared to total density (pQCT), MMR-CLR (RS), and absorbed and fracture energy, at different time points, was performed to this end.

The MDF method enables quantitative assessment of callus development, in contrast to X-rays, which give a qualitative estimation.

In all cases, the result is that MDF monitors bone fracture healing, is reliable, and correlates with all parameters examined, which is not the case for all of the other methods. MDF detects the structural changes due to osteotomy, as well as the bone fracture healing process, in a more sensitive and realistic way compared to all examined parameters, thus enabling bone fracture healing monitoring and detailed objective diagnosis.

Hence, MDF could be a valuable option for clinical use in patients with fractures.

According to our research findings, further human trials are expected to strengthen the above conclusion.

## Data Availability

The original contributions presented in the study are included in the article/Supplementary Material; further inquiries can be directed to the corresponding author.
